# Short Interspersed Element (SINE) Depletion and Long Interspersed Element (LINE) Abundance Are Not Features Universally Required for Imprinting

**DOI:** 10.1371/journal.pone.0018953

**Published:** 2011-04-20

**Authors:** Michael Cowley, Anna de Burca, Ruth B. McCole, Mandeep Chahal, Ghazal Saadat, Rebecca J. Oakey, Reiner Schulz

**Affiliations:** Department of Medical & Molecular Genetics, King's College London, Guy's Hospital, London, United Kingdom; Institut Curie, France

## Abstract

Genomic imprinting is a form of gene dosage regulation in which a gene is expressed from only one of the alleles, in a manner dependent on the parent of origin. The mechanisms governing imprinted gene expression have been investigated in detail and have greatly contributed to our understanding of genome regulation in general. Both DNA sequence features, such as CpG islands, and epigenetic features, such as DNA methylation and non-coding RNAs, play important roles in achieving imprinted expression. However, the relative importance of these factors varies depending on the locus in question. Defining the minimal features that are absolutely required for imprinting would help us to understand how imprinting has evolved mechanistically. Imprinted retrogenes are a subset of imprinted loci that are relatively simple in their genomic organisation, being distinct from large imprinting clusters, and have the potential to be used as tools to address this question. Here, we compare the repeat element content of imprinted retrogene loci with non-imprinted controls that have a similar locus organisation. We observe no significant differences that are conserved between mouse and human, suggesting that the paucity of SINEs and relative abundance of LINEs at imprinted loci reported by others is not a sequence feature universally required for imprinting.

## Introduction

Since the seminal finding that the *Insulin-like growth factor 2* (*Igf2*) gene is subject to genomic imprinting [1], many studies have endeavoured to elucidate the molecular mechanisms responsible for this mode of gene regulation. Epigenetic mechanisms including DNA methylation, histone modifications and non-coding RNAs are now understood to be key players, but genomic sequence and organisation are also important. One of the current challenges of the field is to understand how all of these features integrate to establish and maintain imprinted expression.

A sub-class of imprinted genes have arisen through retrotransposition [2]. In this process, an mRNA molecule becomes associated with the retrotransposition machinery encoded by long interspersed element (LINE)-1 (L1) sequences and is integrated into the genome, producing a duplicate of the original (parent) gene, but lacking introns [3]. In most cases, such events generate pseudogenes, which are defined as genes with sequence similarity to a parent gene but without retention of function [4]. This may occur because the site of integration is not permissive for transcription or the sequence is lacking promoter elements, for example. In rare cases, the new gene provides a selective advantage such that it becomes fixed in a population. Transcription of the gene might occur because of the presence of a cryptic promoter in the sequence or due to integration occurring downstream of an existing promoter. Such genes are termed retrogenes. Several imprinted genes exhibit characteristics of retrogenes. The transcription start sites (TSS) of four of these, *Mcts2*, *Nap1l5*, *U2af1-rs1* and *Inpp5f_v2* [5]–[8], overlap germline differentially methylated regions (gDMRs) that are methylated specifically on the maternal allele (maternal gDMR) and control the parent-of-origin-specific expression of the gene. Other imprinted retrogenes do not possess TSS-associated gDMRs, but retrotransposed into existing imprinted domains presumably accounting for their parent-of-origin-specific expression. *Peg12*, for example, retrotransposed into an imprinted domain on Chr 7 where the gDMR influencing its expression is ∼2.5 Mbp distant at the *Snurf/Snrpn* promoter [9].

**Figure 1:**
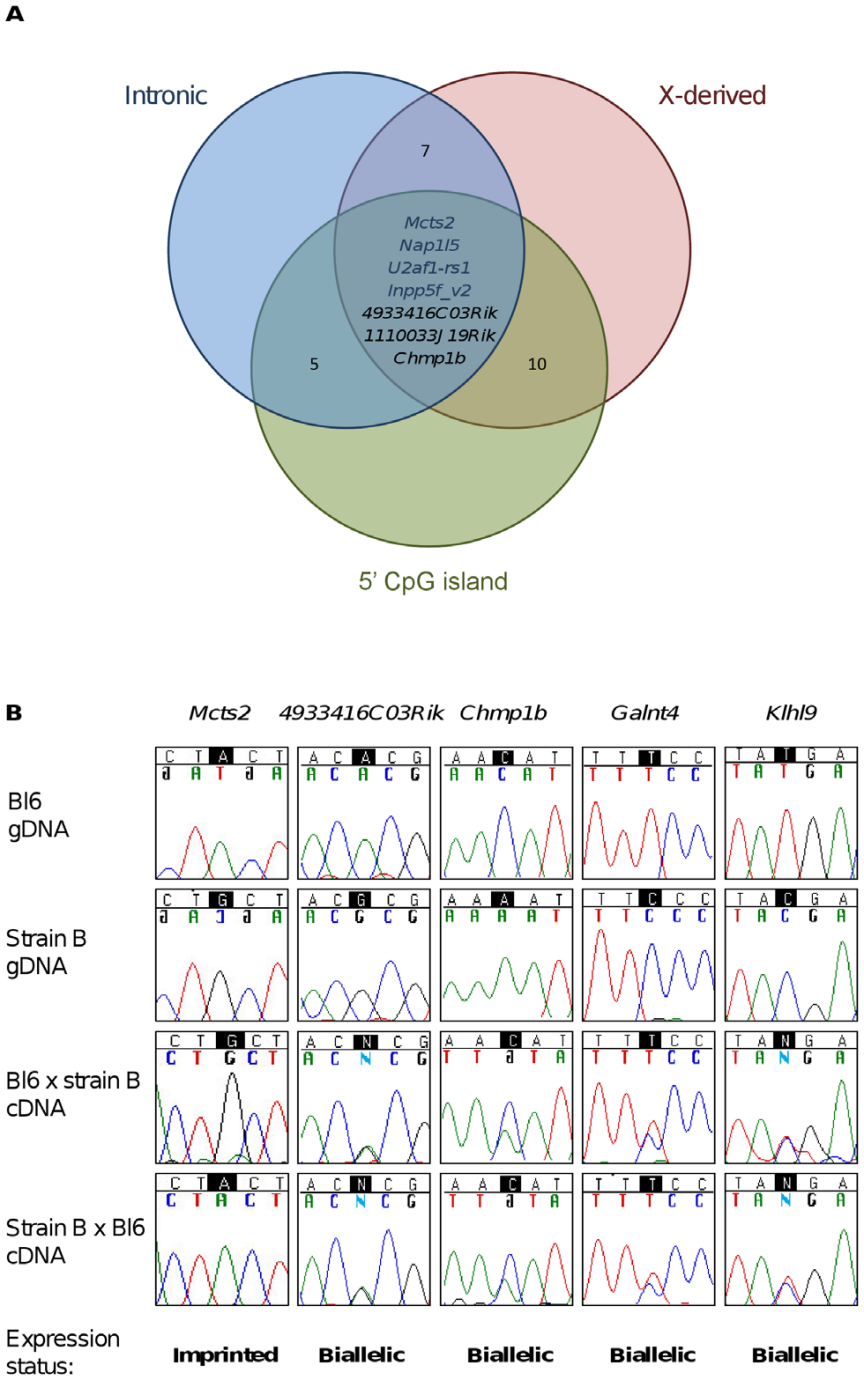
Imprinted and non-imprinted murine retrogenes. (**A**) Four retrogenes expressed from the paternally-derived copy in newborn mouse brain (blue text) share three common features: location within the intron of a host gene, a 5′ CpG island and derivation from a parent gene on Chr X. A further three retrogenes sharing these features are biallelically expressed (black text). Further *in silico* analyses identified additional retrogenes that share two of the three features. The number of genes is indicated at each intersection of the Venn diagram. All were shown to be biallelically expressed (Figure 1b and data not shown). (**B**) Example sequence traces using gene specific primers amplifying from gDNA and newborn brain cDNA over SNPs between Bl6 and another strain (strain B). For *Mcts2*, *4933416C03Rik* and *Klhl9*, strain B is *cast*. For *Chmp1b* and *Galnt4*, strain B is JF1. For the crosses, the maternal strain is presented first. *Mcts2* is imprinted while the others are biallelically expressed. *4933416C03Rik* and *Chmp1b* share all three of the common features, while *Galnt4* and *Klhl9* share only two of the three features (see Table S2).

The four imprinted retrogenes associated with their own maternal gDMRs share three common sequence features (Figure 1a) [5]. Firstly, they are all derived from parent genes on Chr X. This might reflect the bias for autosomal retrogenes to originate from Chr X [10], or imprinting of X-derived retrogenes might be a dosage compensation mechanism for normalising their expression to that of the parent, which would be expressed from a single copy of Chr X [2]. Secondly, each has a CpG island overlapping the TSS. This is relevant since all known maternal gDMRs are promoter-associated CpG-rich regions [11]. Thirdly, they are all positioned within the introns of multi-exonic ‘host’ genes. This may reflect the importance of transcription through imprinted loci to enable germline differential methylation to be established. This has been experimentally demonstrated for the *Gnas* locus, at which ablation of transcripts from the most upstream *Nesp* promoter disrupts oocyte-derived methylation of the *Gnas* DMRs, perturbing imprinted expression [12].

The retrotransposition events which formed the imprinted retrogenes have been dated and modelling of their subsequent evolution has revealed that they have followed distinct evolutionary trajectories [5], [13]. For example, *U2af1-rs1*, derived from the parent gene *U2af1-rs2*, has been under selective pressure to evolve a novel function distinct from that of its parent, whereas *Mcts2*, derived from the parent gene *Mcts1*, has been under purifying selection to retain parent gene function. With this intimate knowledge of how they have arisen and evolved, along with their small size and isolation from large imprinted clusters, these retrogenes are good models with which to study the mechanisms governing imprinting.

A number of studies have reported differences in the prevalence of repeat sequences at imprinted gene loci versus controls [14]–[18]. The consensus is for short interspersed elements (SINEs) to be depleted at imprinted loci, with a moderately increased frequency of LINEs. It is not clear whether this is purely a consequence of imprinting or if repeat element prevalence may be partly responsible or necessary for imprinting. In addition, the methods used by some of the above studies complicate the interpretation of the findings. For example, one study [17] assessed repeat element abundance in variable window sizes, and none of the studies confirmed that the control genes were in fact not imprinted. The tendency for imprinted genes to cluster in the genome relative to non-imprinted genes may also confound the analysis. In the present study, the genomic regions at and around the imprinted retrogenes were examined for repeat element frequency. This was performed in a systematic manner using the most suitable control genes available; specifically, retrogenes with similar locus organisation that are biallelically expressed (i.e., not subject to genomic imprinting). We find no differences in repeat element prevalence at imprinted retrogene loci, suggesting the SINE depletion and LINE abundance previously observed at imprinted loci is not a feature universally required for imprinting.

## Materials and Methods

### Retrogenes

A list of murine retrogenes was obtained from [19]. Retrogenes were classified according to the following features: location within an intron of another gene, derivation from a parent gene on Chr X and the presence of a 5′ CpG island.

### Allele-specific assays

Mouse strains used were C57Bl6 (Bl6), *Mus musculus castaneus* (*cast*) and Japanese Fancy Mouse 1 (JF1). The animals used in this study were wild type, that is, were not genetically modified. They underwent breeding and schedule 1 sacrifice. No procedures were performed on the animals. Therefore, no license was required. RNA was purified from frozen whole brains isolated from day 1 sub-species intercross pups using TRI reagent (Sigma Aldrich). cDNA was synthesised using the SuperScript first strand synthesis kit (Invitrogen). PCR for transcripts of interest and subsequent sequencing was performed over regions containing single nucleotide polymorphisms (SNPs) between mouse strains, as described previously [5]. Primer sequences are available upon request. Sequence data was manipulated using Sequencher. No SNPs could be detected between Bl6 and *cast* or Bl6 and JF1 in the *1110033J19Rik* transcript. Semi-quantitative PCR was performed for this transcript on e18.5 brain cDNA isolated from mice with maternal and paternal uniparental disomy for distal Chr 6 (T77H breakpoint), on which *1110033J19Rik* resides, as well as a wild type control.

### Bioinformatics

Mouse sequences were obtained from build mm9 (July 2007) and human sequences from build hg19 (February 2009) using the UCSC genome browser (http://genome.ucsc.edu/index.html). The gene body was defined as being from the beginning of the CpG island to the base at which the transcript terminates. In the cases of *Inpp5f_v2*/*INPP5F_V2* and the alternative transcript of *RB1*, which splice onto downstream exons of their host genes, the 3′ end of the gene body was defined as the 5′ splice site. Nested windows of 2 kb, 10 kb, 20 kb and 100 kb flanking the gene body were analysed for the presence of repeat elements using the RepeatMasker track of the UCSC genome browser. The mean number of repeat elements are presented with error bars representing the standard error. Statistical analyses, where performed, used Student's T-test.

Murine L1 elements were classified into the F, Tf, Gf and A subfamilies according to their similarity to consensus monomer sequences identified previously [20]–[23]. V subfamily members, although lacking monomers, also possess a unique sequence that was used for identification [24]. Other LINEs were classified according to the RepeatMasker nomenclature. For human L1 elements, scores were assigned according to similarity to the hot L1 consensus sequence, shown previously to be positively correlated with transcription and retrotransposition activity [25].

## Results

We previously used the common features of *Nap1l5*, *U2af1-rs1* and *Inpp5f_v2* to identify *Mcts2* as a novel imprinted retrogene *in silico*, with subsequent experimental validation [5]. In the present study, we extended this work to screen additional retrogenes identified in the mouse genome [19] with the same features for imprinted expression status in the newborn brain. By utilising tissue isolated from mouse sub-species intercrosses, our PCR and sequencing assays provided a qualitative read-out of parental allele contribution. Three additional retrogenes with the three common features were identified *in silico*: *4933416C03Rik*, *Chmp1b* and *1110033J19Rik* (Figure 1a). All three were expressed from both parental alleles, and therefore are not subject to genomic imprinting in the newborn brain (Figure 1b and Figure S1).

The total numbers of SINEs and LINEs in nested windows of 2 kb, 10 kb, 20 kb and 100 kb flanking the gene bodies were assessed for the four imprinted and three non-imprinted retrogenes sharing all three of the common features identified. No consistent differences between imprinted and biallelically expressed retrogenes were observed in any window (Table 1). The mouse genome contains five SINE subfamilies: B1, B2, B4, ID and MIR elements. Only MIR element prevalence differed between imprinted and non-imprinted retrogenes (Table S1). Specifically, MIRs were excluded from the 2 kb window for non-imprinted retrogenes, but this was not the case for the imprinted retrogenes *Mcts2* and *Nap1l5*. However, the small sample sizes utilised in the initial assessment precluded any valid statistical analyses from being performed. We thus sought to increase the statistical power by including additional control (non-imprinted) retrogenes that share two of the three common features (Figure 1a). Specifically, X chromosome-derived retrogenes with 5′ CpG islands but not intronic locations, and intronic retrogenes with 5′ CpG islands but with autosomal parent genes were utilised. Using the approach described above, each of these was assayed for allelic contribution and found to be biallelically expressed (Figure 1b and data not shown). Additionally, the imprinted gene *Nnat* was incorporated into the study to increase the sample size of the imprinted data set. Although the origin of *Nnat* is unclear, and it therefore cannot be defined as a retrogene, it resides within the intron of the host gene *Blcap* and is associated with a 5′ CpG island which is also a gDMR [26]. Both *Nnat* and *Blcap* are imprinted in the mouse brain. The complete list of genes examined is presented in Table S2.

**Table 1:** Total SINEs and LINEs at murine retrogene loci.

Gene	SINEs				LINEs			
	2 kb	10 kb	20 kb	100 kb	2 kb	10 kb	20 kb	100 kb
***Imprinted***								
* Inpp5f_v2*	2	9	18	155	0	2	2	19
* Mcts2*	3	19	27	168	0	0	1	16
* Nap1l5*	1	4	9	52	0	5	7	28
* U2af1-rs1*	5	26	47	165	0	0	4	12
***Biallelic***								
* 4933416C03Rik*	4	12	16	53	0	4	9	59
* Chmp1b*	0	4	14	90	0	2	6	23
* 1110033J19Rik*	2	18	21	74	0	0	1	19

Repeat elements were scored in nested windows from the gene body (see Materials and Methods for definition and further details).

**Figure 2:**
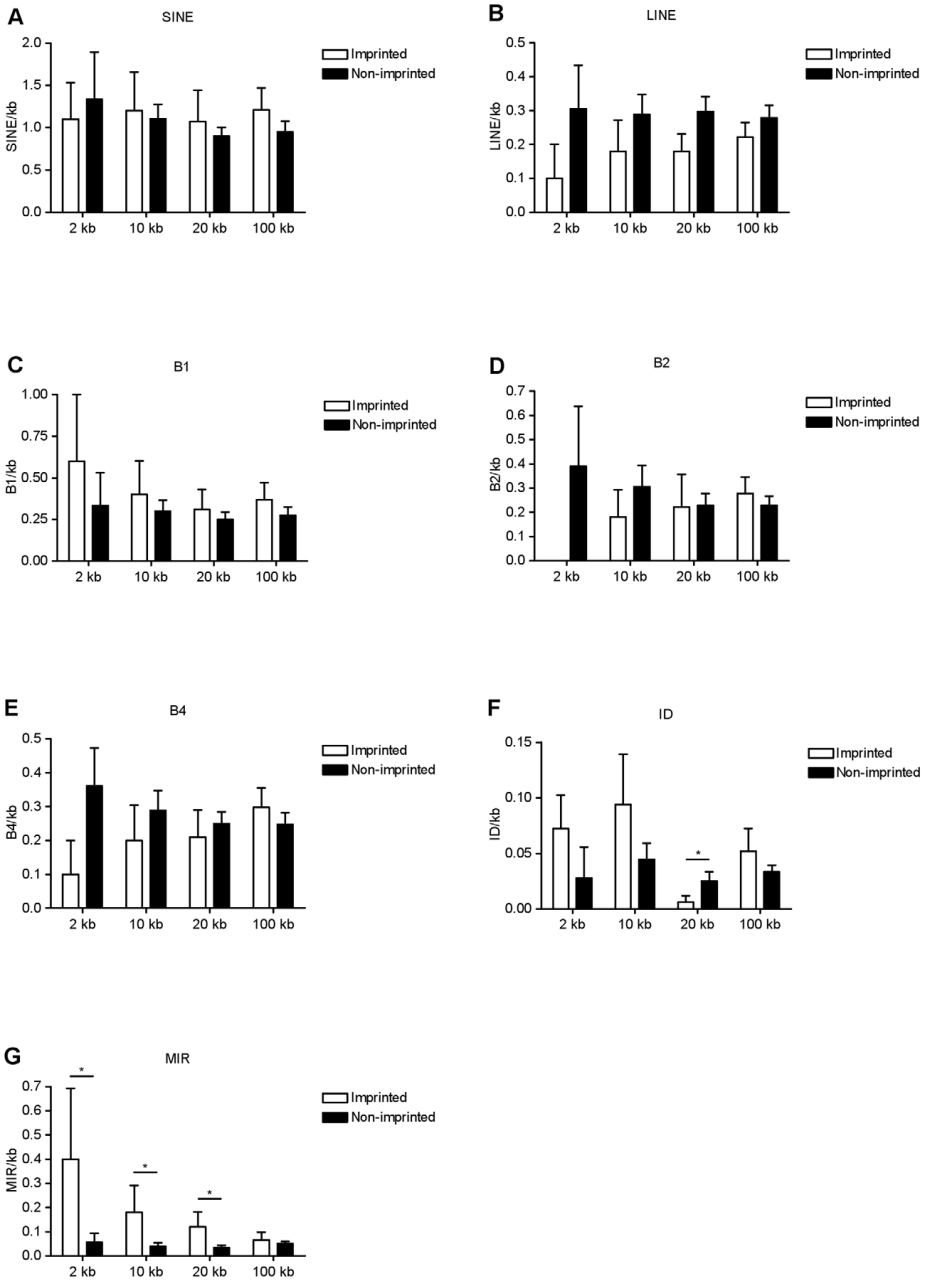
Abundance of SINEs and LINEs at murine retrogene loci. The five imprinted genes were compared with eighteen non-imprinted control retrogenes. The abundance of SINEs (**A**) and LINEs (**B**) is expressed per kb for each nested window. (**C**) **–** (**G**) The abundance of SINE subfamily elements. *p<0.05 by Student's T-test.

Repeat element frequency was assessed for the five imprinted and eighteen control genes using nested windows, as before. SINE abundance did not differ and was consistent across all the windows (Figure 2a). LINEs were relatively less abundant in the 2 kb window flanking imprinted genes, but LINE frequency was consistent across the remaining windows for both datasets (Figure 2b).

Of the SINE subfamilies, the abundance of B1 and B4 elements did not differ between the datasets in any window (Figure 2c and e). B2 elements were excluded from the 2 kb window around imprinted genes while this was not the case for non-imprinted controls, but this did not reach statistical significance with a Student's T-test (Figure 2d). A difference in ID abundance was statistically significant (p<0.05) in the 20 kb window (Figure 2f). MIR elements were relatively abundant near the imprinted genes and this was statistically significant (p<0.05) in the 2 kb, 10 kb and 20 kb windows, with a gradual narrowing of the difference over distance, showing that MIR elements were particularly abundant in the immediate vicinity of the imprinted genes (Figure 2g).

Multiple subfamilies of L1 elements exist in the mouse genome. These are defined by the sequence of repeated monomer units of ∼200 bp within the 5′ untranslated region (UTR) [27]. The Tf, A and Gf subfamilies have active members, meaning they are transcription- and retrotransposition-competent [20]–[21], [23], [27]. Members of the V subfamily, which have no identifiable monomers, and the F subfamily are predominantly inactive [22], [24]. Both inactive and active LINEs play roles in mediating X chromosome inactivation in the mouse [28]. We therefore extended our analysis to include an assessment of L1 subfamily abundance at imprinted retrogenes and *Nnat*, as well as other classes of LINE.

**Figure 3:**
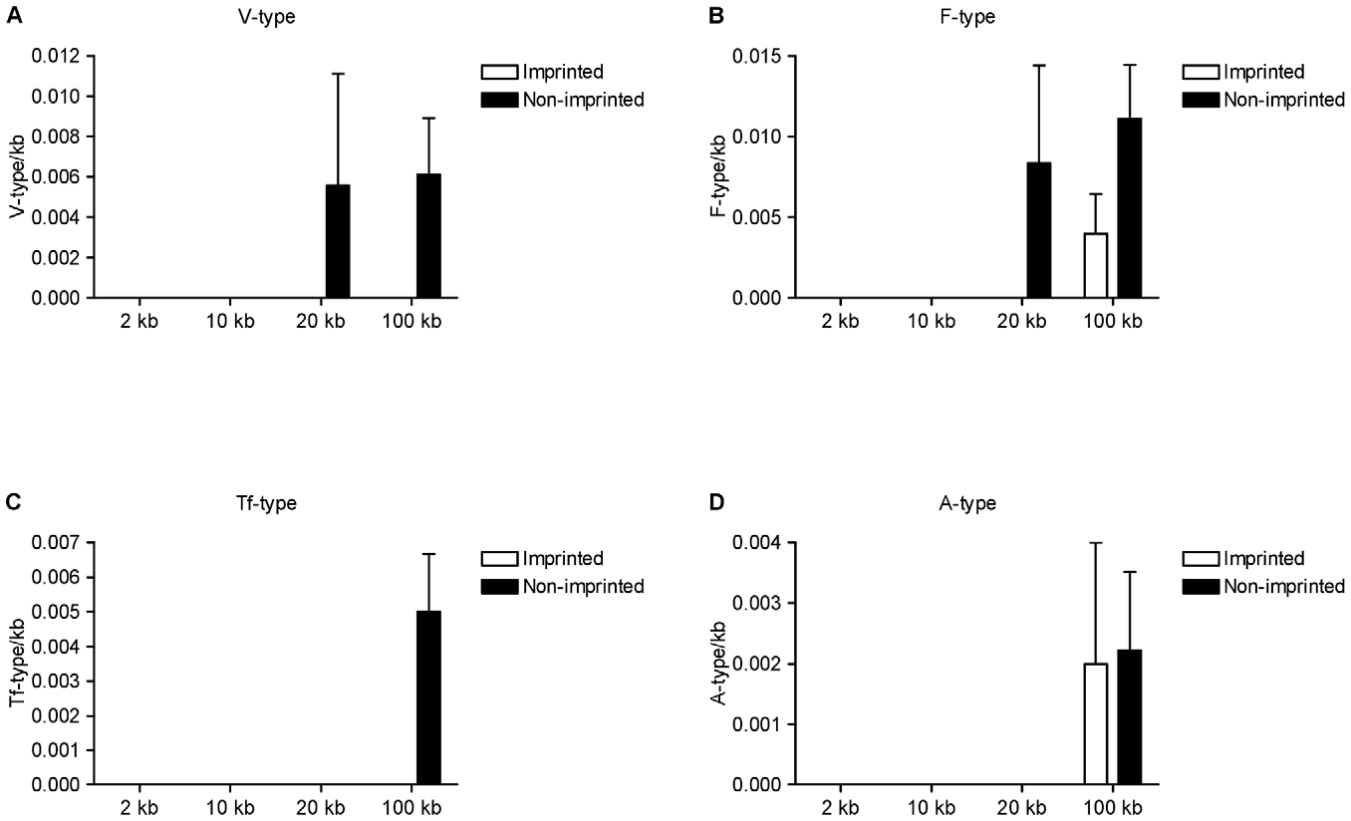
Abundance of L1 subfamilies at murine retrogene loci. The abundance of V (**A**), F (**B**), Tf (**C**) and A (**D**) L1 subfamilies is expressed per kb for each nested window.

Unique sequences were used to classify L1 elements into the Tf, F, A and V subfamilies, as described in Materials and Methods. Gf subfamily members were not identified within the genomic regions examined. No L1 elements of the Tf, F, A and V subfamilies were found within 10 kb flanking imprinted and non-imprinted genes (Figure 3). Elements of the inactive V and F subfamilies were more abundant within 20 kb flanking non-imprinted than imprinted genes but this did not reach statistical significance (Figure 3a and b). Tf and V subfamily elements were more abundant within 100 kb of non-imprinted genes but again this was not significant (Figure 3a and c). The abundance of other LINEs was analysed but no statistically significant differences were observed between the imprinted and non-imprinted gene datasets (Figure S2).

The paucity of SINEs and moderately increased abundance of LINEs at imprinted genes reported in previous studies was not recapitulated in our analysis of imprinted retrogenes and *Nnat*. Only MIR elements were significantly more abundant near the imprinted genes. In order to address the importance of this observation, the human homologues of the imprinted and non-imprinted genes were subjected to the same analysis, where possible. Some retrotransposition events, including that generating the imprinted *U2af1-rs1* gene, occurred after the rodent-primate lineage split [13], [29]. We have previously confirmed conservation of imprinting for *MCTS2*, *NAP1L5* and *INPP5F_V2* [5]. Additionally, an alternative transcript of the human retinoblastoma gene, *RB1*, initiates from a processed pseudogene and is subject to genomic imprinting [30]. Processed pseudogenes are transcriptionally active but do not, themselves, produce a functional protein [4]. Although not X-derived, we included this transcript in the analysis to increase sample size. A list of the human genes screened, consisting of four imprinted genes and ten controls, is presented in Table S3.

**Figure 4:**
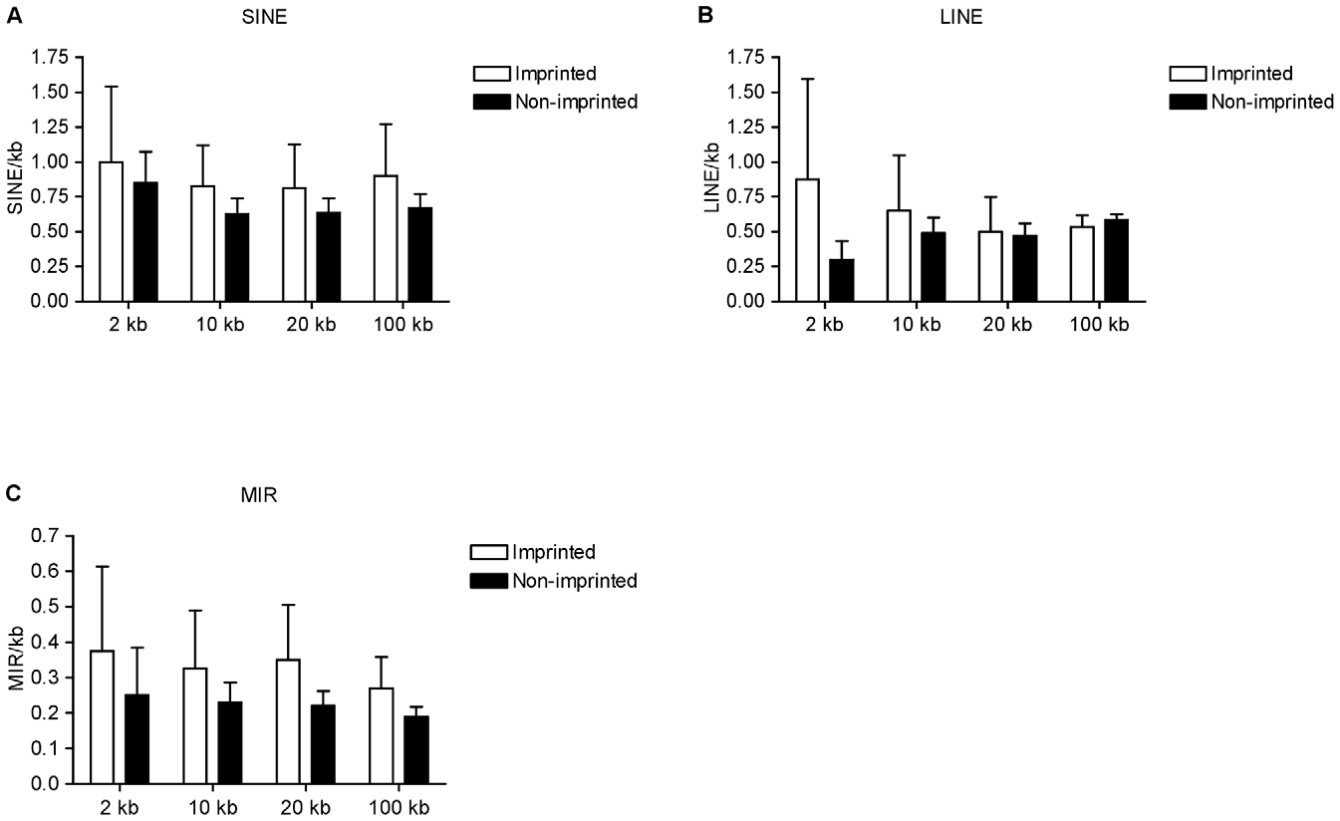
Abundance of SINEs and LINEs at human retrogene loci. The three imprinted retrogenes and one imprinted processed pseudogene were compared with ten controls. (**A**) **and** (**B**) As for Figure 2. (**C**) The abundance of MIR elements.

Comparable to the results observed for mouse, the numbers of SINEs and LINEs in any of the nested windows did not differ significantly between the imprinted and non-imprinted genes (Figure 4a and b). However, the observations for MIR elements observed in mouse were not replicated in human, suggesting that this is not a feature associated with imprinting (Figure 4c). No consistent difference in the abundance of Alu repeats, a primate-specific subgroup of SINEs, at imprinted versus control genes was found (Figure S3).

**Figure 5:**
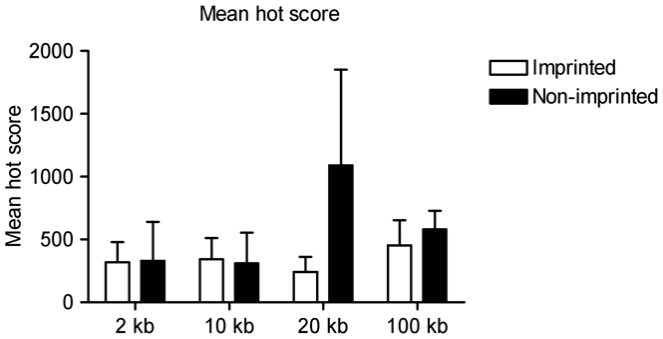
Mean hot score of L1 elements at human retrogene loci. Scores were assigned for L1 elements flanking the analysed genes according to their similarity to the hot L1 consensus sequence [25]. Mean hot scores are presented from the three imprinted retrogenes and one imprinted processed pseudogene compared with ten non-imprinted controls.

In humans, retrotransposition activity of L1 elements is correlated with similarity to a consensus sequence, referred to as the hot L1 consensus [25]. Scores representing similarity to the hot L1 consensus were assigned to L1 elements within nested windows flanking the human gene sets. Mean similarity scores did not differ significantly between imprinted and non-imprinted genes (Figure 5), and we observed a wide range of scores in either case.

## Discussion

Imprinted gene expression is achieved through the interaction of genomic and epigenetic features. Several studies have identified a tendency for SINEs to be relatively rare in the genomic regions flanking imprinted genes, with a moderate increase in the abundance of LINEs [14]–[18]. However, the importance of this relationship has not been fully delineated. For example, is the previously noted paucity of SINEs a necessary genomic feature for imprinting?

Greally, 2002 [14] has argued that SINEs are still able to retrotranspose into imprinted regions, as demonstrated by the presence of young Alu elements in their vicinity, suggesting that SINE integration is an on-going process but occurs at a reduced rate. The paucity of SINEs could thus most probably be accounted for by a reduced accumulation of SINEs near imprinted genes. One reason for this might be to reduce interference of SINE methylation with imprinted gene control. SINEs are methylated to silence their expression and can act as *de novo* methylation centres from which methylation can spread into the surrounding genomic sequence [31]–[32]. Imprinted genes are likely to be particularly sensitive to changes in local methylation patterns because their dosage is tightly controlled through this mechanism. Many imprinted genes play important roles in development and perturbing their expression dosage can have deleterious consequences [33].

Imprinted genes show a tendency to organise into large, complex clusters, with genes sharing regulatory elements in a fashion that appears quite different to that for most non-imprinted genes. The inclusion of imprinted genes from such clusters in previous studies of repeat element abundance may have confounded the analysis. Intragenic regulatory elements may result in the exclusion of SINEs from the region, rather than the imprinted genes *per se*. Further, the gDMRs responsible for controlling imprinting may be located far away from the imprinted gene in question. Previous studies have also indicated that LINEs are at least as abundant around imprinted genes as biallelically expressed genes [14], with one study suggestive of their moderate enrichment in the vicinity of imprinted genes [17]. However, this was observed only at a subset of imprinted genes, specifically those with a G+C content of>40 %. Additionally, this assessment was performed on complete sequences from bacterial and P1 artificial chromosomes (BACs and PACs, respectively) that varied in size from 97.8 kb to 281.0 kb, and was thus not consistent in terms of genomic distance from the imprinted or control gene.

We used imprinted retrogenes to study the relevance of repeat element abundance for imprinting. These genes exhibit similar locus organisation, do not form part of large clusters and are consistently associated with gDMRs at their promoters. Additionally, we used a carefully selected control group of genes with similar properties and confirmed that they are not subject to imprinting in brain, the tissue where all of the imprinted genes in our study exhibit parent-of-origin-specific expression. Our analysis did not reveal any significant differences in LINE or SINE abundance that were consistent between mouse and human. This suggests that LINEs are not genomic features universally required for imprinting. This contrasts with the mechanism of X chromosome inactivation, which requires both silent LINEs to form heterochromatic nuclear compartments within which silent genes reside, and expressed LINEs to facilitate spreading of the silencing mark along the chromosome [28]. In addition, our results indicate that a paucity of SINEs is also not a genomic feature universally required for imprinting.

Both genomic and epigenetic features integrate to control imprinting. Imprinted retrogenes, with their conserved structural organisation, well-characterised evolutionary history and isolation from large imprinted domains, are appropriate models for defining the minimal features required for imprinting. The genomic features we have identified to date that are shared between imprinted retrogenes – a 5′ CpG island, intronic location and derivation from Chr X – are not sufficient for imprinting, as we show in the present study that other retrogenes with these features are biallelically expressed. Our on-going studies are focusing on the importance of other sequence features, such as the origin of the 5′ CpG islands, as well as epigenetic features, such as the presence of binding sites for chromatin modifying proteins, in differentiating imprinted from non-imprinted retrogenes.

## Supporting Information

Figure S1 **Biallelic expression of** ***1110033J19Rik.*** Semi-quantitative RT-PCR using primers specific for the retrogene *1110033J19Rik* was performed from brain cDNA of embryos with maternal and paternal uniparental duplication (UPD) of distal chromosome 6, and a wild type control. Approximately equal expression was detected from all samples, showing that *1110033J19Rik* is biallelically expressed. Negative control samples (no reverse transcriptase, indicated by a – sign) confirm no genomic DNA contamination.
(TIF)

Figure S2 **Abundance of LINE families at murine retrogene loci.**
(TIF)

Figure S3 **Abundance of primate-specific Alu elements at human retrogene loci.** The three imprinted retrogenes and one imprinted processed pseudogene were compared with ten controls. **(A)** Total Alu counts. **(B)** **–** **(E)** The abundance of specific Alu elements. **p < 0.01 by Student's T-test.
(TIF)

Table S1 **Abundance of SINE subfamilies at murine retrogene loci.**
(DOC)

Table S2 **The murine retrogenes used in the study.** The coordinates of the gene bodies, parent gene names and parent gene positions are presented for all the murine genes utilised in the present study. Set 1 refers to genes with all three common features (see Figure 1a). Set 2 refers to genes with 5′ CpG islands and intronic locations, but derived from autosomal parents. Set 3 refers to genes with 5′ CpG islands and Chr X parents, but not intronic locations. Set 4 consists of the imprinted gene *Nnat* which has a 5′ CpG island and an intronic location, but its origin is unclear. Coordinates refer to mouse build mm9 (July 2007).
(DOC)

Table S3 **The human retrogenes used in the study.** The genomic positions are presented for all the human retrogenes utilised in the present study, using the hg19 build (February 2009). *RB1* refers specifically to an alternative transcript of the retinoblastoma gene which is subject to genomic imprinting, and is derived from the parent gene *KIAA0649* on Chr 9 [30]. Thus, this is not a Chr X-derived gene but is helpful in our analyses of the human retrogenes to increase sample size.
(DOC)
