# Supplementary figures and images for: Short Interspersed Element (SINE) Depletion and Long Interspersed Element (LINE) Abundance Are Not Features Universally Required for Imprinting
Source: PLoS One. 2011 Apr 20;6(4):e18953. doi: 10.1371/journal.pone.0018953 (PMC3080381; doi:10.1371/journal.pone.0018953)

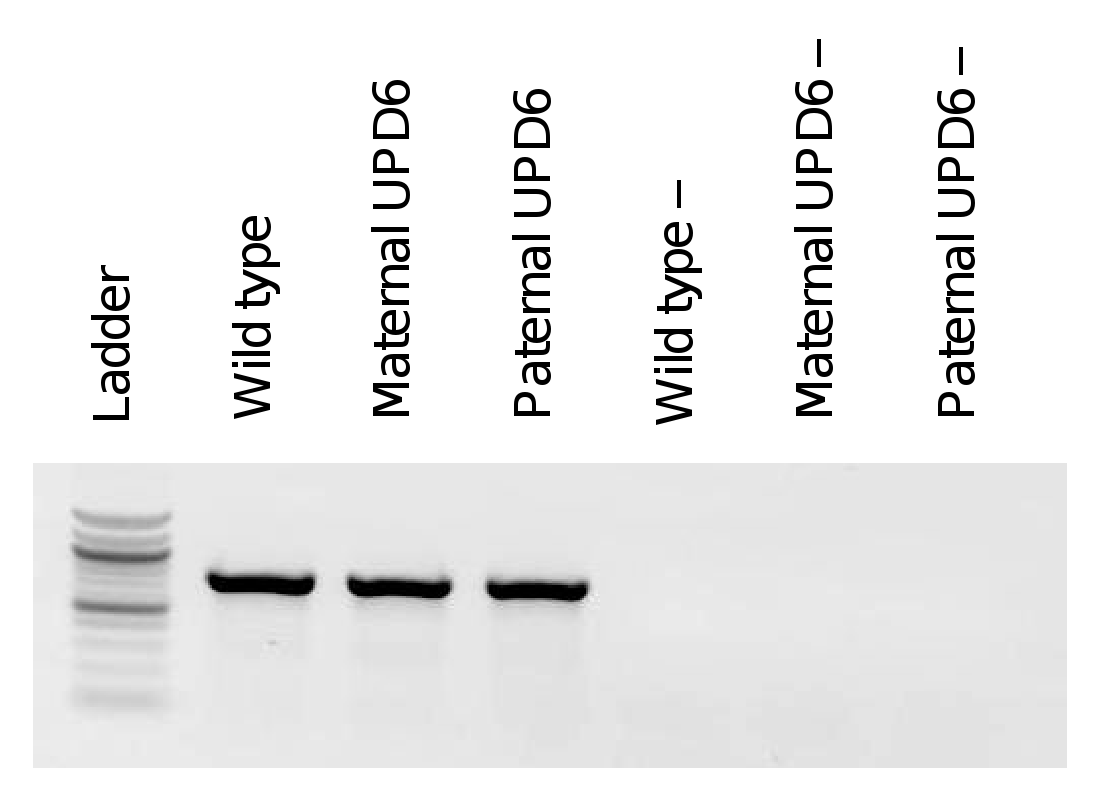

Supplement: Figure S1 — Biallelic expression of 1110033J19Rik. Semi-quantitative RT-PCR using primers specific for the retrogene 1110033J19Rik was performed from brain cDNA of embryos with maternal and paternal uniparental duplication (UPD) of distal chromosome 6, and a wild type control. Approximately equal expression was detected from all samples, showing that 1110033J19Rik is biallelically expressed. Negative control samples (no reverse transcriptase, indicated by a – sign) confirm no genomic DNA contamination. (TIF) [file pone.0018953.s001.tif]

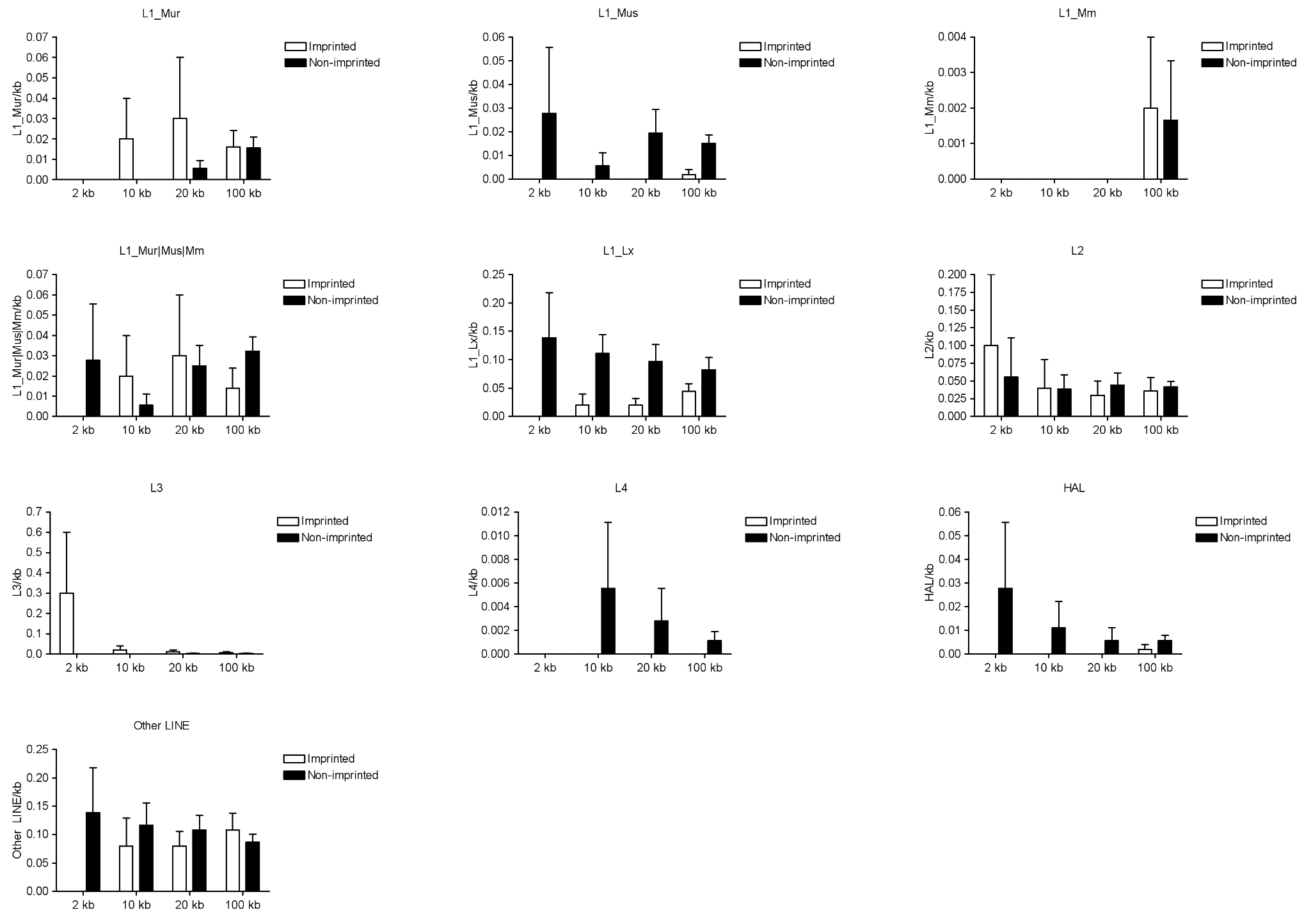

Supplement: Figure S2 — Abundance of LINE families at murine retrogene loci. (TIF) [file pone.0018953.s002.tif]

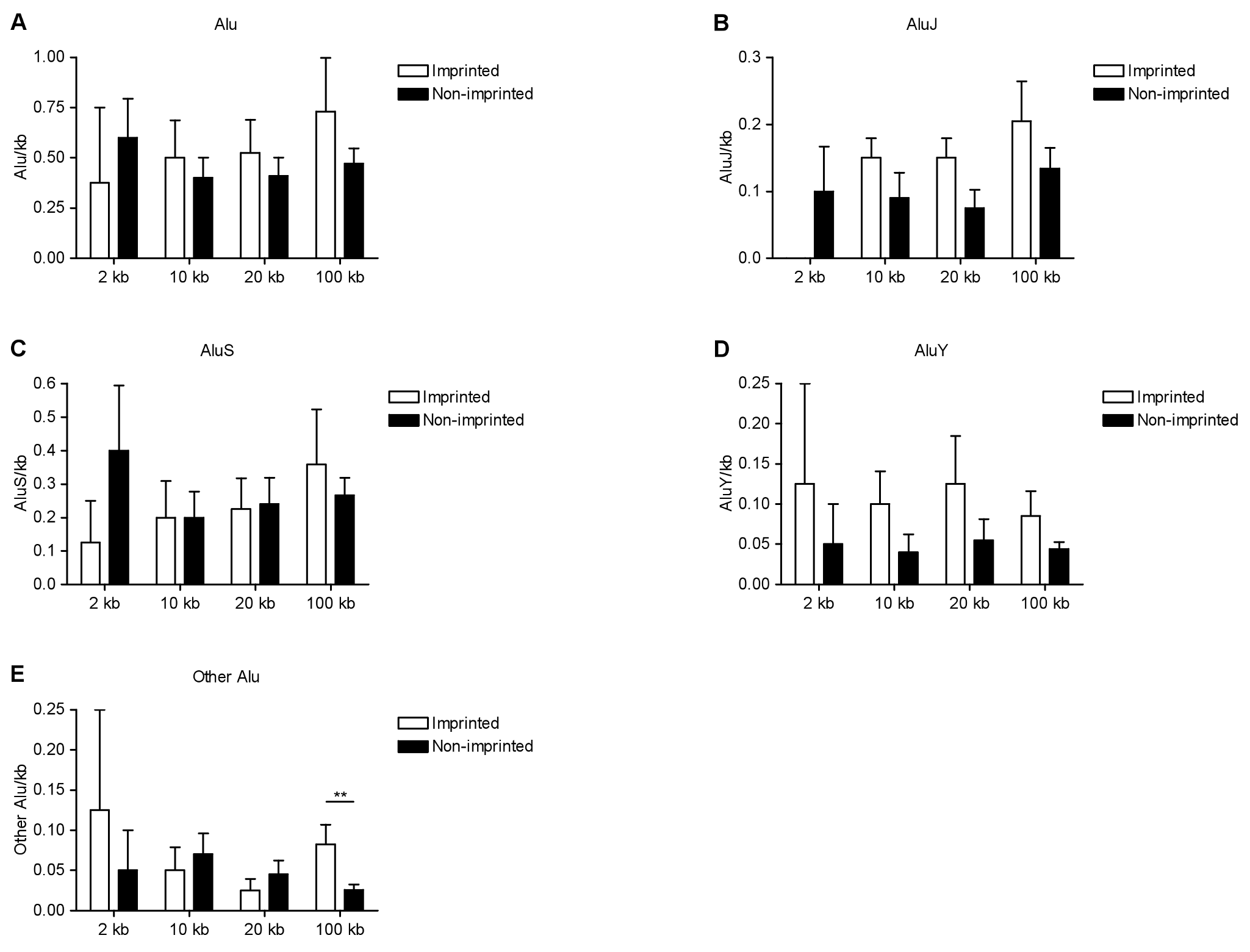

Supplement: Figure S3 — Abundance of primate-specific Alu elements at human retrogene loci. The three imprinted retrogenes and one imprinted processed pseudogene were compared with ten controls. (A) Total Alu counts. (B) – (E) The abundance of specific Alu elements. **p < 0.01 by Student's T-test. (TIF) [file pone.0018953.s003.tif]
