# Supplementary material for: Short Interspersed Element (SINE) Depletion and Long Interspersed Element (LINE) Abundance Are Not Features Universally Required for Imprinting
Source: PLoS One. 2011 Apr 20;6(4):e18953. doi: 10.1371/journal.pone.0018953 (PMC3080381; doi:10.1371/journal.pone.0018953)
Supplement: Table S1 — Abundance of SINE subfamilies at murine retrogene loci. (DOC) [file pone.0018953.s004.doc]

| **Gene** | | **B1 elements** | | | | | | | | **B2 elements** | | | | | | | | **B4 elements** | | | | | | | | **ID elements** | | | | | | | | **MIR elements** | | | | | | | |
| --- | --- | --- | --- | --- | --- | --- | --- | --- | --- | --- | --- | --- | --- | --- | --- | --- | --- | --- | --- | --- | --- | --- | --- | --- | --- | --- | --- | --- | --- | --- | --- | --- | --- | --- | --- | --- | --- | --- | --- | --- | --- |
|  | | 2 kb | | 10 kb | | 20 kb | | 100 kb | | 2 kb | | 10 kb | | 20 kb | | 100 kb | | 2 kb | | 10 kb | | 20 kb | | 100 kb | | 2 kb | | 10 kb | | 20 kb | | 100 kb | | 2 kb | | 10 kb | | 20 kb | | 100 kb | |
| ***Imprinted*** | |  | |  | |  | |  | |  | |  | |  | |  | |  | |  | |  | |  | |  | |  | |  | |  | |  | |  | |  | |  | |
| *Inpp5f_v2* | | 2 | | 6 | | 8 | | 52 | | 0 | | 1 | | 2 | | 29 | | 0 | | 1 | | 3 | | 42 | | 0 | | 1 | | 3 | | 11 | | 0 | | 0 | | 1 | | 1 | |
| *Mcts2* | | 0 | | 2 | | 3 | | 43 | | 0 | | 2 | | 4 | | 36 | | 0 | | 2 | | 5 | | 41 | | 0 | | 2 | | 4 | | 7 | | 3 | | 6 | | 7 | | 19 | |
| *Nap1l5* | | 0 | | 1 | | 3 | | 12 | | 0 | | 0 | | 0 | | 12 | | 0 | | 1 | | 2 | | 14 | | 0 | | 0 | | 0 | | 3 | | 1 | | 2 | | 3 | | 6 | |
| *U2af1-rs1* | | 4 | | 11 | | 15 | | 63 | | 0 | | 6 | | 15 | | 48 | | 1 | | 6 | | 10 | | 33 | | 0 | | 1 | | 2 | | 3 | | 0 | | 0 | | 0 | | 3 | |
| ***Biallelic*** |  | |  | |  | |  | |  | |  | |  | |  | |  | |  | |  | |  | |  | |  | |  | |  | |  | |  | |  | |  | |  |
| *4933416C03Rik* | | 2 | | 3 | | 3 | | 10 | | 0 | | 2 | | 2 | | 6 | | 2 | | 6 | | 8 | | 17 | | 0 | | 0 | | 0 | | 4 | | 0 | | 0 | | 1 | | 10 | |
| *Chmp1b* | | 0 | | 1 | | 2 | | 32 | | 0 | | 0 | | 5 | | 20 | | 0 | | 1 | | 3 | | 13 | | 0 | | 1 | | 1 | | 5 | | 0 | | 1 | | 1 | | 2 | |
| *1110033J19Rik* | | 1 | | 7 | | 8 | | 16 | | 0 | | 2 | | 2 | | 10 | | 1 | | 6 | | 8 | | 31 | | 0 | | 1 | | 1 | | 2 | | 0 | | 2 | | 2 | | 6 | |

Supplementary Table 1
