# Supplementary material for: Short Interspersed Element (SINE) Depletion and Long Interspersed Element (LINE) Abundance Are Not Features Universally Required for Imprinting
Source: PLoS One. 2011 Apr 20;6(4):e18953. doi: 10.1371/journal.pone.0018953 (PMC3080381; doi:10.1371/journal.pone.0018953)
Supplement: Table S2 — The murine retrogenes used in the study. The coordinates of the gene bodies, parent gene names and parent gene positions are presented for all the murine genes utilised in the present study. Set 1 refers to genes with all three common features (see Figure 1a). Set 2 refers to genes with 5′ CpG islands and intronic locations, but derived from autosomal parents. Set 3 refers to genes with 5′ CpG islands and Chr X parents, but not intronic locations. Set 4 consists of the imprinted gene Nnat which has a 5′ CpG island and an intronic location, but its origin is unclear. Coordinates refer to mouse build mm9 (July 2007). (DOC) [file pone.0018953.s005.doc]

Supplementary Table 2

| **Retrogene** | **Gene body coordinates** | **Parent gene** | **Parent gene coordinates** |
| --- | --- | --- | --- |
| ***Set 1*** |  |  |  |
| *Inpp5f_v2* | chr7:135831484-135832762 | *BC028317*/*Vma21* | chrX:69062171-69066395 |
| *Mcts2* | chr2:152512546-152513678 | *Mcts1* | chrX:35953836-35966699 |
| *Nap1l5* | chr6:58857141-58855227 | unknown | unknown |
| *U2af1-rs1* | chr11:22871975-22874908 | *U2af1-rs2* | chrX:160388321-160396530 |
| *4933416C03Rik* | chr10:115551397-115548745 | *Taf7l* | chrX:130994659-131011029 |
| *Chmp1b* | chr18:67364924-67366655 | *2610002M06Rik* | chrX:104983149-105003241 |
| *1110033J19Rik* | chr6:148303077-148304118 | *Rps4x* | chrX:99380370-99383710 |
| ***Set 2*** |  |  |  |
| *Dnajb3* | chr1:90102374-90101310 | *Dnajb6* | chr5:30062530-30112998 |
| *Oxct2a* | chr4:123000858-122999118 | *Oxct1* | chr15:3976429-4103962 |
| *Galnt4* | chr10:98569416-98575881 | *Galnt12* | chr4:47104826-47135914 |
| *Oxct2b* | chr4:122793503-122795243 | *Oxct1* | chr15:3976429-4103962 |
| *BC087907* | chr15:73640358-73639988 | *Ndufb4* | chr16:37647689-37654454 |
| ***Set 3*** |  |  |  |
| *BC025076* | chr11:62461524-62474991 | *Tmem32* | chrX:53838690-53851123 |
| *Klhl26* | chr8:73000964-72974127 | *Klhl13* | chrX:22796398-22892333 |
| *Klhl9* | chr4:88368577-88364196 | *Klhl13* | chrX:22796398-22892333 |
| *D1Pas1* | chr1:188791269-188794506 | *Ddx3x* | chrX:12858165-12869740 |
| *Pdha2* | chr3:140874853-140872968 | *Pdha1* | chrX:156560152-156576268 |
| *1700012H05Rik* | chr7:114352692-114354430 | *Rbmx* | chrX:54639536-54646199 |
| *4921504I05Rik* | chr13:21560501-21558968 | *Nkap* | chrX:34666812-34690741 |
| *2500001K11Rik* | chr1:121422403-121424803 | unknown | unknown |
| *1700029P11Rik* | chr15:81810890-81811991 | *Ndufb11* | chrX:20192453-20194680 |
| *Btg1* | chr10:96079119-96082261 | mRNA AK132937 | chrX:35367086-35367570 |
| ***Set 4*** |  |  |  |
| *Nnat* | chr2:157385787-157388255 | origin unclear |  |
