# Supplementary material for: Short Interspersed Element (SINE) Depletion and Long Interspersed Element (LINE) Abundance Are Not Features Universally Required for Imprinting
Source: PLoS One. 2011 Apr 20;6(4):e18953. doi: 10.1371/journal.pone.0018953 (PMC3080381; doi:10.1371/journal.pone.0018953)
Supplement: Table S3 — The human retrogenes used in the study. The genomic positions are presented for all the human retrogenes utilised in the present study, using the hg19 build (February 2009). RB1 refers specifically to an alternative transcript of the retinoblastoma gene which is subject to genomic imprinting, and is derived from the parent gene KIAA0649 on Chr 9 [30]. Thus, this is not a Chr X-derived gene but is helpful in our analyses of the human retrogenes to increase sample size. (DOC) [file pone.0018953.s006.doc]

Supplementary Table 3

| **Retrogene** | **Gene body coordinates** |
| --- | --- |
| *INPP5F_V2* | chr10:121577530-121579052 |
| *MCTS2* | chr20:30135077-30136019 |
| *NAP1L5* | chr4:89617068-89619053 |
| *RB1* | chr13:48890958-48895555 |
| *CHMP1B* | chr18:11851210-11854447 |
| *DNAJB3* | chr2:234651396-234652720 |
| *OXCT2* | chr1:40235198-40237189 |
| *GALNT4* | chr12:89913191-89919903 |
| *KLHL9* | chr9:21331019-12335559 |
| BC049659 | chr6:3849272-3851023 |
| *RBMXL2* | chr11:7110133-7112379 |
| *4921504I05Rik* | chr6:28226980-28228736 |
| *2500001K11Rik* | chr2:120978855-120981450 |
| *BTG1* | chr12:92540333-92534054 |
